# 378. Cost-effectiveness of an adaptive platform trial design compared to sequential conventional clinical trials for comparative drug evaluations in bloodstream infections: a simulation study

**DOI:** 10.1093/ofid/ofad500.448

**Published:** 2023-11-27

**Authors:** Sean W X Ong, Nick Daneman, Steven Y C Tong, David Naimark

**Affiliations:** University of Toronto, Toronto, Ontario, Canada; Sunnybrook Health Sciences Centre, University of Toronto, Toronto, Ontario, Canada; Doherty Department University of Melbourne, Peter Doherty Institute for Infection and Immunity, Melbourne, Victoria, Australia; Sunnybrook Health Sciences Centre, Toronto, Ontario, Canada

## Abstract

**Background:**

Adaptive platform trials (APTs) have become increasingly popular in recent years in infectious diseases research. However, few studies have compared APT design against conventional randomized clinical trial (RCT) design from a cost-effectiveness standpoint. We aimed to evaluate the cost-effectiveness of APT versus conventional RCTs and quantify the trade-offs involved in choosing between these designs.

**Methods:**

We conducted a model-based economic evaluation using a two-level, hierarchical simulation model comparing two strategies: (1) APT comparing three drugs simultaneously against a single control group, and (2) three sequential 2-arm parallel group conventional RCTs (Fig 1). Cost inputs were obtained from a recently completed conventional RCT studying bloodstream infections (BSI) and a recently launched APT for Gram-negative BSI (Table 1). 1000 Monte Carlo 2nd order iterations were performed to simulate 1000 RCTs to determine empirical Type I and II error rates across several scenario analyses.

**Figure 1:**
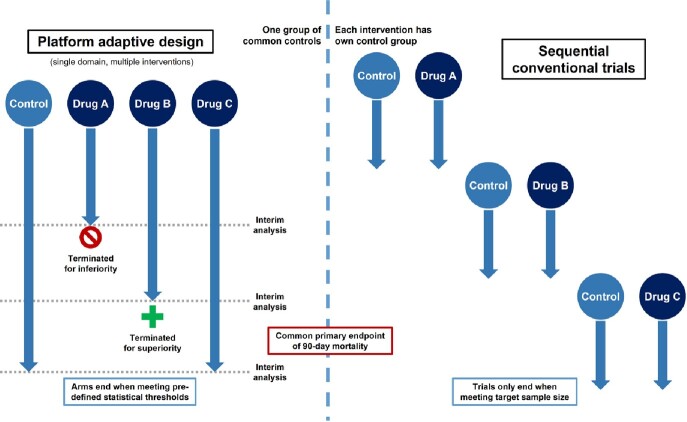
Schematic illustrating adaptive platform trial and conventional clinical trial design used in model.

**Table 1:**
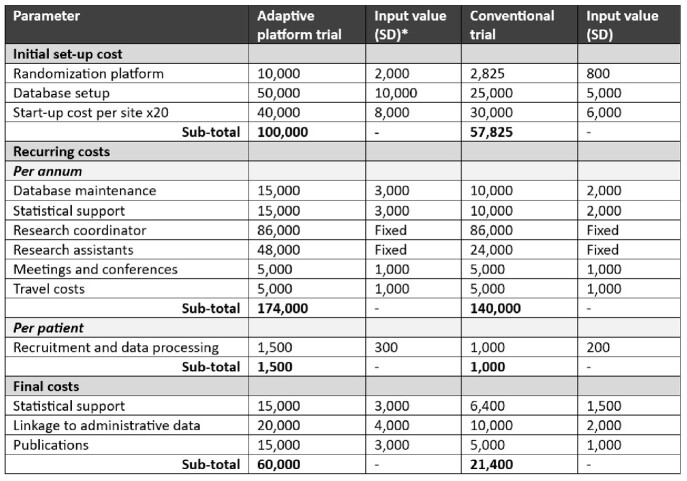
Cost inputs for adaptive platform trial and conventional clinical trial design. SD = standard deviation. All costs are stated in Canadian dollars. * Input standard deviations stated if costs input as gamma distributions.

**Results:**

In the base case analysis where a less stringent interim analysis stopping rule was used, and the drugs being tested were effective, APT design was associated with lower cost ($5,368,000 vs $8,655,000), shorter duration (135 vs 242 weeks), and lower mean type II error (0.086 vs 0.213) (Table 2). However, results were highly sensitive to different scenario analyses where more stringent stopping rules were applied or if the tested drugs had no true effect. Effect sizes were less precise and on average were over-estimated with APT design (Fig 2). Type I error rates were also consistently higher with the APT strategy (mean error rates of 0.20 and 0.077 using liberal and strict χ^2^_crit_ values of 3.841 and 6.635 respectively) compared to conventional design (fixed at 0.05 by design) (Fig 3).

**Table 2:**
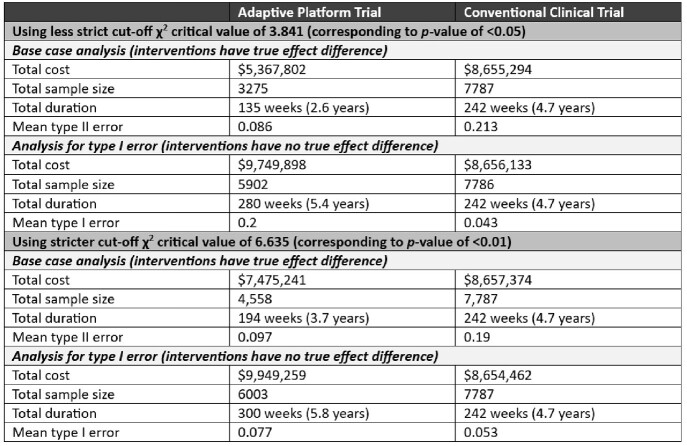
Results of base case and scenario analyses. All cost stated are in Canadian dollars.

**Figure 2:**
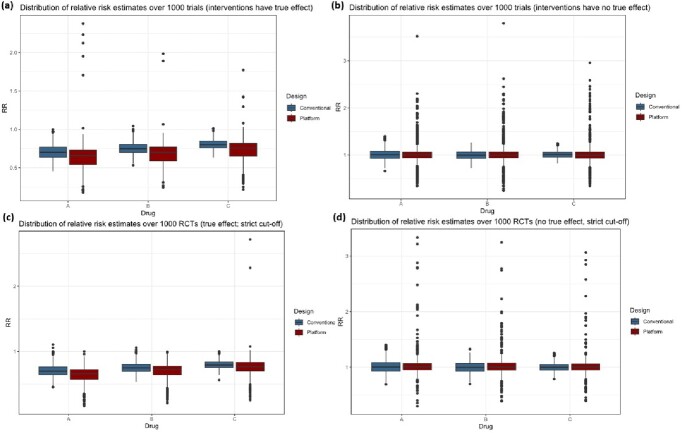
Distribution of relative risk over 1000 RCTs associated with APT and conventional RCT strategies for base case and scenario analyses. (a) Distribution of RR when drugs have true effect (RR of 0.7, 0.75, and 0.8 respectively). Adaptive platform trial design was associated with less precise estimates (wider ranges) and on average over-estimated the effect size. (b) Distribution of RR when drugs have no true effect (RR of 1.0 for all three drugs). (c) and (d) represent the same scenarios as (a) and (b) but with stricter interim analysis cut-offs (χ2 critical values of 6.635 vs 3.841; corresponding to p-value of 0.01 vs 0.05 respectively.

**Figure 3:**
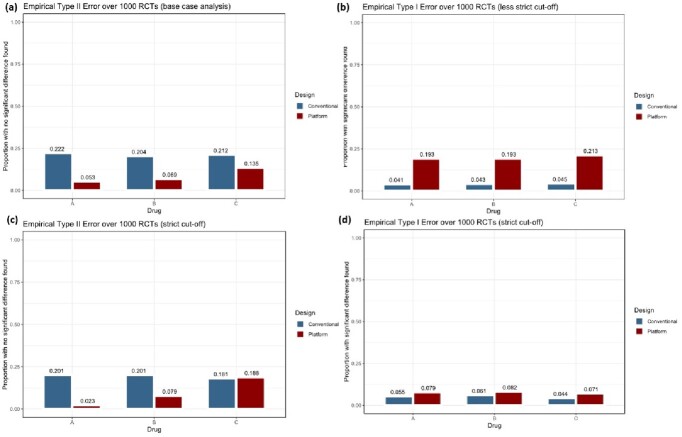
Empirical type I and type II error associated with APT and conventional RCT strategies for base case and scenario analyses. (a) Empirical type II error associated with APT and conventional trials, calculated by determining the proportion of 1000 RCTs where no significant difference was concluded when drugs were simulated to have a true effect. Conventional trial design had type II error rates of about 0.20 by design (in sample size calculation). APT design was associated with lower type II error rates. The same effect was seen in (c) where stricter cut-off values for interim analysis was used. (b) Empirical type I error associated with APT and conventional trials, calculated by determining the proportion of 1000 RCTs where a significant difference was concluded when drugs were simulated to have no true effect (RR of 1.0). APT was associated with a consistently higher type I error rate, even when a stricter interim analysis cut-off was used (d).

**Conclusion:**

We show a proof-of-concept that simulation methods can be used to compare APT and conventional RCT designs for trial planning purposes. Neither strategy was consistently superior in terms of cost-effectiveness. Trade-offs in cost, sample size, and error rates are highly scenario dependent. Choice of trial design should depend on multiple variables, including the study question, probability of efficacy of the drug, and priorities of the investigator (Table 3).

**Table 3:**
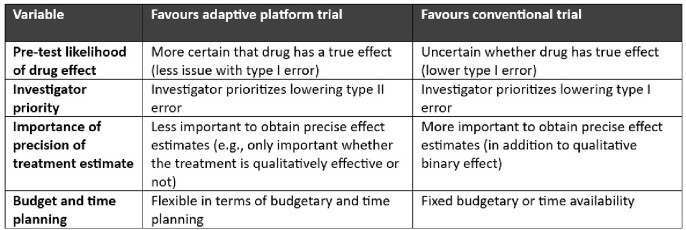
Factors affecting choice of RCT design.

**Disclosures:**

**All Authors**: No reported disclosures

